# Association between Sense of Coherence and Mental Health in Caregivers of Older Adults

**DOI:** 10.3390/ijerph16203800

**Published:** 2019-10-09

**Authors:** Catalina López-Martínez, Natalia Serrano-Ortega, Sara Moreno-Cámara, Rafael del-Pino-Casado

**Affiliations:** 1 Department of Nursing, Faculty of Health Sciences, University of Jaén, 23071 Jáen, Spain; cmartine@ujaen.es (C.L.-M.); smcamara@ujaen.es (S.M.-C.); 2 Surgical nursing, Reina Sofia University Hospital, 14004 Córdoba, Spain; nataliaserrano@hotmail.com

**Keywords:** caregivers, older people, sense of coherence, anxiety, depression, subjective burden, quality of life

## Abstract

The purpose of this study was to analyze association between sense of coherence and perceived burden, anxiety, depression, and quality of life in caregivers of older adults. A cross-sectional study was carried out with a probabilistic sample of 132 caregivers of older relatives from the regions of Jaén, Spain. The measures assessed were sense of coherence (Life Orientation Questionnaire), subjective burden (Caregiver Strain Index of Robinson), anxiety and depression (Goldberg Scale), and quality of life (Health Questionnaire SF-12). The main analyses included bivariate analysis using Pearson’s correlation coefficient and multivariate analysis through canonical correlation analysis. Our findings show that the sense of coherence explained 50.8% of the variance shared between subjective burden, anxiety, depression, and quality of life. We highlighted manageability as the variable within the dimensions of the sense of coherence with the greatest participation in the model. The sense of coherence may be an important protective factor for the mental health of the caregiver of dependent elderly relatives.

## 1. Introduction

Most countries are experiencing an increase in the number and proportion of elderly people. In this scenario, older people increase the likelihood of disabilities and need the support of family, friends or long-term care services [1]. In Europe, informal care for older people covers 80% of care needs [2].

Caregivers have a higher prevalence of mental health problems as a result of caregiving activity [3]. The main emotional problems presented by people who care for a dependent relative are anxiety, depression, and subjective burden [4]. According to the literature, these problems are due to the stress that caregivers may experience in caregiving, and the way they perceive, live, and manage. This issue has been previously described by the stress model of Lazarus et al. [5], in which the strategies and resources used by people in stress situations influence the consequences of stress. 

Caring for a dependent relative can also produce positive effects, including improved health and perceived well-being, which can be measured by evaluating quality of life [6]. According to the World Health Organization (WHO), quality of life is defined as “the way in which the individual perceives the place he occupies in the cultural environment and in the value system in which he lives, as well as in relation to his objectives, expectations, criteria and concerns” [7]. The evaluation of the quality of life is based on a wide range of criteria, including positive aspects of life [7].

Among the different factors related to the positive and negative effects of caring for dependent elderly relatives, the sense of coherence (SOC) has received significant attention. SOC is a concept developed by Antonovsky within the salutogenetics theory. This theory is oriented towards the promotion of health, seeing the healthy part of the person instead of focusing on the disease [8,9]. SOC refers to the orientation a person has to understand a specific situation, the ability to use available resources, and to take into account the meaning of their challenges [10]. In addition, this theory identifies a series of human resources and conditions called general resources of resistance (RGRs). These resources include money, experience, knowledge, healthy habits, commitment, self-esteem, social support, cultural capital, the version of life, intelligence, and traditions of society [10]. The concept of SOC is composed of three dimensions: meaningfulness, comprehensibility, and manageability. Meaningfulness (the motivational dimension) refers to “the extent to which one feels that life has an emotional meaning, that at least some of the problems faced in life a face are worth commitment and dedication, and are seen as challenges rather than only as burden” [11]; comprehensibility (the cognitive dimension) refers to “the extent to which one perceives internal and external stimuli as rationally understandable, and as information that is orderly, coherent, clear, structured rather than noise—that is, chaotic disordered, random, unexpected, and unexplained” [11]; and manageability (the instrumental or behavioral dimension) is defined as “the degree to which one feels that there are resources at one’s disposal that can be used to meet the requirements of the stimuli one is bombarded by” [11].

Several studies relate SOC with the positive and negative effects of caring for a dependent elderly relative. Hsiao et al. [12] related SOC with the mental component of the quality of life (positive association) and with subjective burden (negative association) in caregivers of relatives with mental health problems. Mizuno et al. [13] revealed that SOC was accompanied by a better quality of life. Jaracz et al. [14] found a negative association of SOC with subjective burden, anxiety, and depression in caregivers of family members with stroke. Orgeta et al. [15] showed that SOC was associated with lower anxiety and depression in caregivers of family members with dementia. No studies have been found that simultaneously analyze the effect of SOC on a set of positive and negative emotional consequences. Such an analysis would give a greater perspective on the predictive capacity of SOC regarding caregivers’ mental health. 

We have identified works that analyze the effect of one or more factors on a set of caregiving consequences independent of the SOC. Myaskovsky et al. [16] analyzed the effect of different coping strategies on the components of quality of life in caregivers of relatives with lung transplant. Additionally, Rodríguez-Sánchez et al. [17] studied the effect of family function on different dimensions of quality of life. 

These previous studies used canonical correlation analysis. Other studies not related to family care utilize this methodology of analysis to analyze the relationships between the dimensions of SOC and beliefs [18] or personality [19]. The canonical correlation analysis, developed by Hotelling [20], is a technique to explore the relation between two sets of multiple covariates by measuring the linear relationship between them [21]. 

The aim of this study was to analyze the relationship between the dimensions of SOC and subjective burden, anxiety, depression, and the mental component of quality of life in caregivers of dependent elderly relatives through canonical correlation analysis.

## 2. Materials and Methods 

### 2.1. Design, Sample, and Settings

The present study is cross-sectional. The study population consisted of caregivers of elderly relatives in the districts of La Loma, Las Villas, Sierra Mágina, and Sierra de Segura de Jaén in the Jaén-Nordeste Health District (Andalusia, Spain). The sampling frame consisted of a census of 4645 caregivers of elderly dependents in health centers of the District. The caregivers were selected through a systematic random sampling of census data. A “dependent” was considered to be a person who relied on the caregiver for at least one activity of daily life (instrumental or basic).

The sample size was calculated to detect statistical association with a correlation coefficient of at least 0.21, with 95% safety and 80% power. The calculated sample size was 132 participants. This sample size meets the criteria of Osterlind et al. [22] and Crespín Elías [23] regarding avoidance of “overfitting” the data in a multivariate analysis. These authors recommended at least ten subjects per measured variable.

### 2.2. Data Collection

The data were collected throughout 2015 via interviews at the care-recipient’s home, carried out by nurses with at least ten years of experience and with specific training in the care of family caregivers. These nurses held a training session to manage the tools used in the study.

Contact with potential participants was carried out by their family nurses or case management nurses. Participants were contacted by phone; during the call, the characteristics of the study were briefly explained, and an interview was arranged if the person agreed to participate. A pilot study was conducted (*N* = 20) with the intention of evaluating the procedures and making the appropriate improvements in data collection methods. Before the interview, all participants were informed about the confidentiality of the collected data and were instructed to sign an informed consent. This study was approved by the Research Ethics Committee of Jaén.

### 2.3. Ethical Considerations

Before the interview, all participants were informed about the confidentiality of the collected data and were instructed to sign an informed consent. The study was conducted in accordance with the Declaration of Helsinki and the protocol was approved by the Ethics Committee of Jaén (2706201306).

### 2.4. Measurements

#### 2.4.1. Sociodemographic Variables

The data collected were caregiver age, gender, kinship with the caregiver recipient, and duration of caregiving; care recipient age, gender, and cause and type of dependence.

#### 2.4.2. Sense of Coherence

SOC was measured using the Life Orientation Questionnaire developed by Antonovsky [24]. This scale is an abbreviated version of SOC-29 with 13 items. The questionnaire consists of three dimensions: meaningfulness, manageability, and comprehensibility. The items are measured with a Likert scale of 7 points (1—Very often, 7—Rarely or never). Scores range from 13 to 91 for the global questionnaire, from 4 to 28 for the meaningfulness dimension, from 4 to 28 for the manageability dimension, and from 5 to 35 for comprehensibility. The questionnaire has been validated in the Spanish population by Virués-Ortega et al. [25], with adequate clinimetric properties (Cronbach’s alpha = 0.76). In our study, the internal consistency measured by Cronbach’s alpha was 0.79 for the global questionnaire, 0.7 for the meaningfulness dimension, 0.6 for the manageability dimension, and 0.65 for the comprehensibility dimension.

#### 2.4.3. Subjective Burden

Burden data were collected using the Caregiver Strain Index developed by Robinson [26]; this study specifically used the Spanish version of Moral Serrano et al. [27]. The Index is composed of thirteen dichotomous true/false questions, with the answer “true” scoring one point and total scores ranging from zero to thirteen points. López Alonso et al. [28] validated this scale in Spanish caregivers of dependents with a Cronbach’s alpha of 0.808. In our study, the internal consistency had a Cronbach’s alpha of 0.776.

#### 2.4.4. Anxiety and Depression

These variables were collected using the Goldberg Anxiety and Depression Scale [29]. This scale is composed of two subscales (anxiety and depression), each containing nine dichotomous yes/no questions. Affirmative answers score one point for a total of nine points for each subscale; scores are proportional to the caregiver’s level of anxiety or depression. This scale was validated in the Spanish population by Montón Franco et al. [30] with good results (Cronbach’s alpha for anxiety of 0.83 and 0.84 for depression). In our study, internal consistency had a Cronbach’s alpha of 0.83 for anxiety and 0.84 for depression.

#### 2.4.5. Quality of Life

Quality of life data were collected with the SF-12 Health Questionnaire [31]. Twelve questions were evaluated using the Likert scale. The scale ranges from 0 to 100, proportional to the state of health. The questionnaire can be evaluated by eight first order dimensions (physical function, social function, physical role, emotional role, mental health, vitality, corporal pain, and general health) or by two second-order dimensions (physical component and mental component) [32]. In the present study, the mental component was used. The internal consistency of the mental component was 0.80 (Cronbach’s alpha).

### 2.5. Statistical Analysis

Initially, a descriptive analysis of mean and standard deviation for each measurement was performed. This was followed by a bivariate analysis using correlation coefficients. Finally, multivariate analysis with canonical correlation was performed to measure the degree of linear relationship between the two resulting sets of variables and estimate the amount of variance shared between the dimensions of SOC and the rest of the variables. Next, the canonical loads of each of the variables were analyzed with the intention of identifying patterns of relationship between the variables.

We identified the assumptions that must be fulfilled for our analysis of canonical correlations. These assumptions are those of the General Linear Model [21]: linearity, analyzed with the quantile-quantile (Q-Q) graphs; multicollinearity, analyzed by tolerance and variance inflation factor; homoscedasticity, analyzed by the Durbin-Watson test; multivariate normality, measured by the Kolmogorov-Smirnov test and the Shapiro-Wilk test multivariate; and the absence of extreme points, evaluated by a box diagram and the matrix scatter plot. The level of statistical significance was set at 5%. The calculations were made with the statistical package IBM SPSS Statistics v. 24 (IBM Corp, Armonk, NY, USA).

## 3. Results

### 3.1. Descriptive Analysis

The sample consisted of 132 caregivers. The sociodemographic profile of the participants is shown in Table 1. The caregivers who participated were mainly women with an average age of 56 years; most were daughters and had cared for an average of nine years. The care recipients were mostly women who had an average age of 85 years. Care recipients also had predominantly chronic or physical problems. The SOC mean was 63.59 (SD = 13.64); the mean values obtained in each of the dimensions were 21.29 (5.06) for meaningfulness, 19.20 (5.07) for manageability, and 23.11 (6.08) for comprehensibility. Anxiety and depression scores had values of 4.06 (SD = 2.93) and 2.91 (SD = 2.74) respectively. The mental component of quality of life presented a mean of 34.38 (9.09).

Assumptions of normality were evaluated in order to verify the performance of the statistical tests. The assumptions of the General Linear Model were confirmed with the exception of the depression variable, which required transformation by the method of Box et al. [33] to attain normality. Durbin–Watson values were between 1.973 and 2.116, and the tolerance values were greater than 0.57. 

### 3.2. Multivariate Analysis

In the preliminary correlation analysis, negative associations were obtained between meaningfulness and subjective overload (r = −0.438) and depressive symptoms (r = −0.532), manageability was negatively associated with anxiety (R = −0.551) and positively associated with the mental component of quality of life (r = 0.376 **), and comprehensibility was negatively associated with depressive symptoms (r = −0.520 **) (Supplementary 1).

The analysis evaluated the multivariate-shared relationship between set one, which included the variables belonging to the SOC dimensions and set two, which included the variables belonging to the positive and negative consequences of caregiving.

The canonical correlation model yielded three functions with square canonical correlations of 0.416, 0.146 and 0.012 for each function. The model was statistically significant with a Wilks’s lambda (λ) of 0.492, F (12, 312.49) = 8.015, and *p* < 0.001. Wilks’s λ indicates the amount of variance not shared among the sets of variables, so 1- λ will give us the overall effect for the whole model. This result can be interpreted in the same way as an R^2^ in multiple regression. In the set of the three canonical functions, we observed a Wilks’s λ of 0.508, which suggests that the complete model explained approximately 50.8% of the variance shared between the sets of variables. 

In the analysis, the reduction of dimensions showed the hierarchical level of the functions showing that the first two functions were statistically significant. For function 2 we also observed statistical significance with a Wilks’s λ of 0.842, F (6238.00) = 3.552, and *p* < 0.001. Function 3 did not have statistical significance, with a Wilks’s λ of 0.987, and did not provide more data.

Given the effects for each function, only the first two functions were considered important in the context of this study, explaining 41.60% and 14.66% of shared variance, respectively. Function 3 explained only 1.29% of the remaining variance in the sets of variables after separating it from the previous functions.

**Figure 1:**
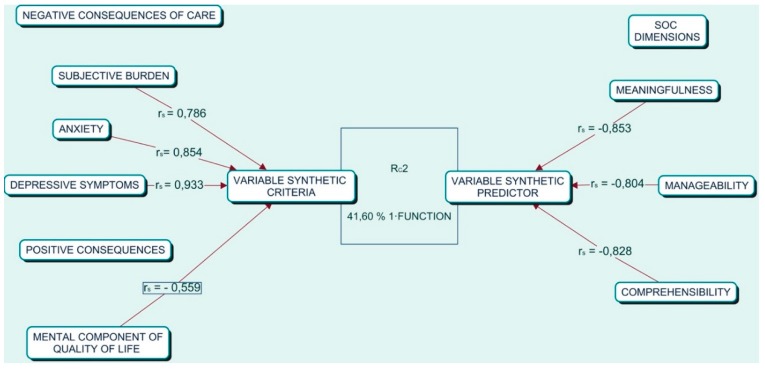
Conceptual model of canonical correlation.

In Table 2, we provide the standardized function and correlations of canonical structure or canonical loads (r_s_) for the two first functions. We also provide the square structure coefficients (r_s_^2^) and communality coefficients (h^2^) for each of the variables through both functions. The coefficients of function 1 (Figure 1) demonstrate the relevance of the dimensions of SOC as important given their high contributions, with the meaningfulness and comprehensibility variables being particularly vital despite their short distance. We observed that all the coefficients have the same sign, so all were positively related.

With regard to the consequence variables in function 1, depression, anxiety, and burden were the variables with the most important contribution to the model, with the mental component of quality of life taking a secondary position (Figure 1). The structure coefficients of previous variables (depression, anxiety, and burden) had the same sign, indicating that they are positively related. The mental component of the quality of life had a sign opposite to these variables, suggesting an inverse relationship to the negative consequences of care. These results are also sustained by the square structure coefficient. These relationships can be seen in Figure 1.

In Table 2, the coefficients in function 2 indicate that the only relevant variable for the SOC was manageability, although less than in function one. In the set of consequences, anxiety and the mental component of quality of life are the predominant variables. Data showed that these variables are related in an inverse manner.

When observing the structure coefficients for the whole function, it was apparent that manageability was inversely related to anxiety and directly related to the mental component of the quality of life. If we observe the communality coefficients, we can see that in each set, there is a variable with a more important contribution than the rest; manageability and anxiety were the most outstanding.

## 4. Discussion

In the present study, SOC was directly related to the mental component of quality of life and inversely related to burden, anxiety, and depression in caregivers of older relatives. In addition, canonical analysis showed that SOC explained 50.8% of the variance of previous caregiving consequences. 

Our results match those of studies in the general population that relate SOC with better emotional health [34,35] and quality of life [36]. Our results also coincide with those of studies in caregivers that relate SOC with quality of life [37,38,39], subjective burden [14,40], anxiety [15], and depression [14,15]. Our findings also match with those of a systematic review [41] that relates SOC with subjective burden, anxiety, and depression.

According to the literature, the negative consequences of a caregiving activity on caregivers’ emotional health and quality of life are due to the experienced stress and the way in which this stress is perceived and managed by caregivers [12,42,43]. Our results are consistent with those who argue that SOC allows better management of stressful events, making these events more understandable, meaningful, and manageable [10]. In our study, among the three dimensions of SOC, manageability is the most important. These results match those of Sutter et al. [44] and show that the ability to evaluate stressful events as being manageable can have great importance in controlling stressors.

The abovementioned studies that analyze SOC and caregiving consequences use nonprobabilistic samples and do not simultaneously analyze the effect of SOC on a set of positive and negative emotional consequences. Our findings build upon previous results by analyzing and quantifying the common influence of SOC on several indicators of the emotional health of caregivers using probabilistic sampling. Other strengths of the present study were sample size calculations a priori performed, the utilization of experienced examiners, a pilot testing of the examination protocol, and the usage of well accepted and internationally used measures with previous and own validation. Moreover, the characteristics of the sample analyzed in this study are similar to those of the Spanish Institute for the Elderly and Social Services (IMSERSO in Spanish) survey on care for the elderly in Spanish homes [31], which is representative at a national level. Therefore, this sample could be considered highly representative of the caregivers of dependent elderly people in Spain. 

The present study has the limitation of being cross-sectional; it is only possible to assess associations and relationships between the variables without the ability to draw causal conclusions from the data. Our model predicted only about 50% of the variation. Recent systematic reviews [45,46] have found that anxiety, depression, and subjective burden were related to caregiver characteristics (e.g., demographic –gender–, family relationships, social support, and coping) and care-recipient characteristics (e.g., functional capacity and cognitive impairment). Thus, variance not predicted by our model could be due to previous caregiver or care-recipient characteristics.

Because caregiving consequences included in the present study can approximate the mental health state of the person, our findings support that SOC may be a good predictor of the mental health of persons caring for a dependent elderly relative. Thus, SOC could be used as a screening tool that enables giving early attention to those caregivers who have the greatest risk of deteriorating mental health. 

Moreover, because several studies have shown that SOC increases through interventions that focus on coping [47], problem solving [48], cognitive therapy [49], or lifestyle interventions [50], our findings support the implementation of interventions addressed to enhance SOC. Strengthening the SOC of caregivers has been a topic of great interest in recent years [51]. While large, randomized controlled trial studies are needed to fully investigate this issue, data suggest that early detection of low SOC could allow implementation of interventions which enhance SOC.

## 5. Conclusions

Sense of coherence is associated with several measures of mental health among caregivers of older relatives. Thus, it can be speculated that sense of coherence may work as a screening tool of mental health among caregivers of older adults and that SOC-strengthening interventions aimed at reducing the risk of the negative emotional consequences of caregiving might be necessary. Whether sense of coherence is a measure that changes over time similarly with other mental health measures warrants further investigations.

## Figures and Tables

**Table 1:** Descriptive measures of the analyzed sample.

Caregivers		
		*n* (%) or M (SD) [Range]
Age (years)		56.3 (11.8) [23–89]
Gender	Women	114 (86.4%)
	Men	18 (13.6%)
Kinship tie	Spouse	17 (12.9%)
	Offspring	98 (74%)
	Political children	6 (4.5%)
	Other	11 (8.3%)
Duration (years)		9.19 (7.879) [0.67–47]
Sense of coherence		63.6 (13.6) [13–91]
Meaningfulness		21.29 (5.059) [4–28]
Manageability		19.20 (5.068) [4–28]
Comprehensibility		23.11 (6.084) [5–35]
Subjective burden		5.39 (3.165) [0–13]
Anxiety		4.06 (2.931) [0–9]
Depressive symptoms		2.91 (2.742) [0–9]
Quality of life (mental component)		34.38 (9.090) [0–100]
**Care Recipients**		
		***n* (%) or M (SD) [Range]**
Age (in years)		85.30 (6.16) [67–100]
Gender	Women	100 (75.8%)
	Men	32 (24.2%)
Type of dependence	Physical	79 (59.8%)
	Mixed	52 (39.4%)
	Psychic	1 (0.8%)
Cause of dependence	Chronic problems	62 (47%)
	Physical impairment	39 (29.6%)
	Cognitive impairment	15 (11.4%)
	Terminal patient	10 (7.6%)
	Stroke	4 (3.1%)

Note: M: mean; SD: standard deviation.

**Table 2:** Canonical results for sense of coherence (SOC) dimensions and positive and negative consequences for each function.

Variables	Function 1			Function 2			h^2^
	Coef.	r_s_	r_s_^2^ (%)	Coef.	r_s_	r_s_^2^ (%)	
Meaningfulness	−0.519	−0.853	72.76	0.718	0.400	16	88.76
Manageability	−0.366	−0.804	64.64	−1.179	−0.594	35.28	99.92
Comprehensibility	−0.317	−0.828	68.55	0.406	0.030	0.09	68.64
R_C_^2^			41.60			14.66	
Subjective burden	0.329	0.786	61.77	−0.386	−0.061	0.37	62.14
Anxiety	0.177	0.854	72.93	1.433	0.474	22.46	95.39
Depressive symptom	0.572	0.933	87.04	−1.166	−0.170	2.89	89.93
Quality of Life/mental component	−0.101	−0.559	31.24	−0.300	−0.331	10.95	42.19

Notes: Coef.: standardized canonical coefficient; r_s_: structure coefficient or canonical load; r_s_^2^: square structure coefficient; h^2^: communality coefficient; Rc^2^: square canonical correlation coefficient.

## Supplementary Materials

The following are available online at https://www.mdpi.com/1660-4601/16/20/3800/s1, Appendix 1: Pearson‘s correlation coefficients of study variables.
