# Supplementary material for: Association between Sense of Coherence and Mental Health in Caregivers of Older Adults
Source: Int J Environ Res Public Health. 2019 Oct 9;16(20):3800. doi: 10.3390/ijerph16203800 (PMC6843852; doi:10.3390/ijerph16203800)
Supplement: Supplementary file 1 [file ijerph-16-03800-s001.pdf]

International Journal of Environmental Research and Public Health

MDPI

Appendix 1. Pearson`s correlation coefficients of study variables.

**Variables**	**1**	**2**	**3**	**4**	**5**	**6**	**7**	**8**
1 Sense of coherence	1							
2 Meaningfulness	0.792**	1						
3 Manageability	0.830**	0.463**	1					
4 Comprehensibility	0.892**	0.558**	0.644**	1				
5 Subjective burden	-0.469**	-0.438**	-0.386**	-0.360**	1			
6 Anxiety	-0.555**	-0.402**	-0.551**	-0.451**	0.610**	1		
7 Depressive symptoms	-0.595**	-0.532**	-0.440**	-0.520**	0.532**	0.746**	1	
8 Quality of life (mental component)	0.523**	0.246**	0.376**	0.297**	-0.309**	-0.489**	-0.443**	1

Notes: * = p ≤ .05; ** = p ≤ .01; M: mean; SD: standard deviation.
